# Experimental and Simulation Investigation of Nd Additions on As-Cast Microstructure and Precipitate Development in Mg–Nd System Alloys

**DOI:** 10.3390/ma15072535

**Published:** 2022-03-30

**Authors:** Xuewei Yan, Bin Su, Xuemei Yang, Qingdong Xu, Xiaopeng Zhang, Jing Wang, Zhenhua Wen

**Affiliations:** 1 School of Aero Engine, Zhengzhou University of Aeronautics, Zhengzhou 450046, China; yanxuewei@zua.edu.cn; 2 Institute of Materials, China Academy of Engineering Physics, Jiangyou 621700, China; 138268104xu@163.com (Q.X.); saviorggg@163.com (X.Z.); wangjing7@caep.cn (J.W.); 3 School of Aerospace Engineering, Zhengzhou University of Aeronautics, Zhengzhou 450046, China; yangxuemei@zua.edu.cn

**Keywords:** Mg–Nd alloy, microstructure, precipitate, eutectic growth, modelling and simulation

## Abstract

The microstructure and precipitate evolution of as-cast Mg–Nd alloys with different contents of Nd was investigated via experimental and simulation methods. The research showed that the as-cast microstructure of Mg–Nd alloy consisted of *α*-Mg dendrites and the intermetallic phases. A metastable *β* phase precipitated, followed by *α*-Mg dendrites that could be confirmed as Mg_12_Nd by X-ray diffraction (XRD) analysis. The amount of *β*-Mg_12_Nd presented a rising trend with increasing Nd additions. In addition, the tertiary phase was also observed in as-cast Mg–Nd alloy when Nd content was greater than 3 wt.%, which precipitated from the oversaturated *α*-Mg matrix. The tertiary phase should be *β*_1_-Mg_3_Nd, which is also a metastable phase with a face-centered cubic lattice. However, it is a pity that the tertiary phase was not detected by the XRD technique. Moreover, an effective cellular automaton (CA) model was explored and applied to simulate the time-dependent *α*-Mg/*β*_1_-Mg_3_Nd eutectic growth. The simulated results of *α*-Mg/*β*_1_-Mg_3_Nd eutectic growth in Mg-3Nd presented that the growth of *α*-Mg dendrites was accompanied by the nucleation and growth of *β*_1_-Mg_3_Nd precipitates and eventually formed a eutectic structure. The eutectic morphologies for Mg–Nd system alloys with different Nd contents were also simulated using the proposed model, and the results revealed that *α*-Mg dendrite was a refinement, and the amount of *α*-Mg/*β*_1_-Mg_3_Nd eutectic was promoted, with increasing Nd content.

## 1. Introduction

Magnesium (Mg) and its alloys have received increasing attention in recent years due to their low-density, high specific strength and excellent creep resistance [1,2,3]. The abundance of magnesium in the earth’s crust and the potential low-cost make it a viable alternative to steels or aluminum alloys for structural components in the automotive, aerospace and electronics industries [4,5]. However, Mg and Mg alloys suffer from poor mechanical properties, corrosion resistance and high-temperature strength because the hexagonal close-packed (HCP) structure leads to insufficient slip systems, strong basal texture and poor plasticity [6,7,8]. Improvements to these deficient properties of Mg alloys could be achieved through alloying and the consequent heat treatment, in particular through adding alloyed elements to fine grains and increasing the amount of precipitates and solid solution [9,10].

Recently, many studies have focused on the modification of the alloy elements, with a special emphasis on additions to improve the mechanical properties of Mg alloys. Alloys such as Mg–Al [11], Mg–Zn [12], Mg–Zr [13] and Mg–RE (rare earth) [14] system alloys have been developed towards the high creep resistance. Among them, Mg–RE alloys were attractive due to their remarkable age-hardening response, excellent creep resistance and good formability [15]. Specifically, Mg–Gd [16], Mg–Y [17] and Mg–Nd [18] are the most common heat-resistant Mg alloys. Over the past few decades, more and more research information concerning Mg–Nd alloy both experimentally and theoretically have been extensively reported in the literature. Yan et al. [19] investigated the creep behavior of Mg-2Nd alloy under different temperature and applied stress conditions and found that this alloy exhibited good creep resistance due to both solution hardening and, especially, precipitation hardening. Hantzsche et al. [20] systematically evaluated the effect of Ce, Nd and Y additions on the microstructure and texture development in Mg–RE alloys, and the authors concluded that the amount of the RE addition required for sufficient texture weakening was connected with the solid solubility of the respective element. Zhu et al. [21] investigated the relationship between the microstructure and creep resistance in Mg–RE alloys, and the authors reckoned that the strengthening of *α*-Mg matrix by solid solution and/or precipitation was more important than grain boundary reinforcement by intermetallic phases for the creep resistance of Mg–RE alloys. Liu et al. [22] studied the effect of Al content on the microstructure and mechanical properties of as-cast Mg–5Nd alloys, and the results indicated that Al additions could significantly lead to the grain refinement of Mg–5Nd alloy and ascribed the greatly improved mechanical properties to the refined grains and secondary phase strengthening. It is well known that microstructures are the strategic link between a material’s process and performance, and, thus, further investigation of the detailed interaction between the microstructure and the properties of Mg–Nd alloys is of great importance to control the desirable microstructure and improve the performance.

In addition, some recent reports [23,24,25] displayed that the high strength and good creep resistance of Mg–Nd alloy resulted from the formation of fine precipitates of metastable phases. Thus, a deep understanding of precipitated behavior in Mg–Nd alloy may provide a capability for the rational design of composition and processes to improve the performance of Mg–Nd alloy. However, the precipitation sequence of Mg–Nd alloys remains controversial and have been the topic of a number of experimental and computational studies. Saito et al. [26] observed the microstructure of precipitates formed in an Mg–0.5Nd alloy by high-angle annular detector dark-field scanning transmission electron microscopy, and the results indicated that the precipitation sequence could be Mg-solution → GP-zone → *β*′ (Mg_7_Nd, orthorhombic) → *β*_1_ (Mg_3_Nd, FCC). Liu et al. [27] developed a phase-field model to examine the heterogeneous nucleation of *β*_1_ precipitates in Mg–0.5 at.% Nd alloy, and the authors found that *β*_1_ precipitated as ultra-thin laths with abnormally large aspect ratios under the influence of the stress field of a screw dislocation. Zhu et al. [28] presented the formation of linear-chain distribution features of precipitates in Mg–Nd alloys, and the results showed that the configuration consists of bamboo trunks of *β*_1_ variants and trunk connections of a hexagonal lattice *β*_2_ phase. Natarajan et al. [29] reported a combined computational and experimental study of phase stability and precipitation in Mg–Nd alloys, and the results supported the following precipitation sequence for binary Mg–Nd alloys: SSSS (supersaturated solid solutions) → GP zones (Guinier–Preston zones, hexagons) → *β*′′′ → *β*_1_ (Mg_3_Nd) → *β* (Mg_12_Nd) → *βe* (Mg_41_Nd_5_). Considering the studies mentioned above, precipitation has long been considered one of the most effective approaches for manipulating the kinetics and homogeneity in Mg–Nd alloys, and the related research has made significant progress over the past few years. However, the microstructure and precipitate controls in Mg–Nd alloys are still challenges due to the complex precipitation behavior, especially in the as-cast alloys. Therefore, a further study is needed for the satisfied microstructures and accurate precipitates in Mg–Nd system alloys.

The aim of the present work was to investigate the microstructure and precipitate development in as-cast Mg–Nd alloys using combined experimental and computational methods during the solidification process. The influence of different Nd additions on the microstructure and precipitate morphologies was examined using an optical microscope (OM) (ZEISS, Oberkochen, Baden Wurttemberg, Germany) and a scanning electron microscope (SEM) (FEI Company, Hillsboro, ORE, USA). Chemical composition and phase characterization were also performed by the energy-dispersive spectroscopy (EDS) and X-ray diffraction (XRD) techniques. In addition, an effective model for predicting eutectic evolution behavior in Mg–Nd system alloys was established, and the eutectic growth and the final morphologies were simulated to analyze the effects of the Nd additions.

## 2. Experimental Procedure and Simulation Method

### 2.1. Materials and Experimental Methods

The experimental materials employed in this work were Mg–*x* Nd (*x* = 1, 2, 3, 4, 5, 6 and 7, wt.%) system alloys, which were prepared in a 60 KW level electric resistance furnace (Henan Sante Furnace Technology Co., Ltd., Luoyang, Henan, China) under the protection of a mixed gas atmosphere of 1% SF_6_ and 99% CO_2_. The high-purity Mg (99.98 wt.%) (Luoyang Maige Magnesium Industry Co., Ltd., Luoyang, Henan, China) was preheated to 150 °C for several minutes to displace the moisture and then melted at 750 °C. When the pure Mg melted sufficiently, the temperature was elevated to 780 °C and moderate Mg-20 wt.% Nd master alloy was added. After being stirred to remove slag for 10 min, the melting alloy then stood for 30 min at 750 °C. Subsequently, the power was cut off, the melting alloy cooled to ~720 °C, and it was then poured into a mild steel mold (80 mm in diameter, preheated to ~300 °C), which was covered with a mold release agent (boron nitride) (Hefei Kejing Material Technology Co., Ltd., Hefei, Anhui, China). The filled mold then cooled in the air until the melting alloy was completely solidified. The as-cast ingots were machined and polished, and the chemical compositions were measured using energy-dispersive X-ray fluorescence (EDXRF). The results as well as the designed compositions are listed in Table 1.

The samples for microstructural analysis were ground, mechanically polished and then etched with a mixture of 5% HNO_3_ and 95% ethanol. The microstructures were characterized by an optical microscope equipped with a digital camera, and the precipitated phases were observed using a focused ion beam-scanning electron microscope (FIB-SEM, FEI Helios Nanolab 600i) (FEI Company, Hillsboro, ORE, USA) at 15 kV with a working distance of 4 mm. The precipitates were qualitatively analyzed using X-ray diffraction (XRD, TDF-3000, Dandong Tongda Science & Technology Co., Ltd., Dandong, China), and a microanalysis was performed on the different phases in the microstructure using energy-dispersive X-ray spectroscopy (EDS) equipped with an EDS detector (EDAX Inc., Philadelphia, PA, USA) for elemental analysis. The volume fraction and average size of the secondary phase were statistically measured using OM micrographs captured from different regions and calculated by Image-Pro Plus software (Version 6.0, Media Cybernetics, Inc., Los Angeles, CA, USA). Partial thermophysical parameters (thermal conductivity and specific heat) were measured at different temperatures, and the results provided access to the data needed in the simulations.

### 2.2. Model Description and Simulation Parameters

**Figure 1:**
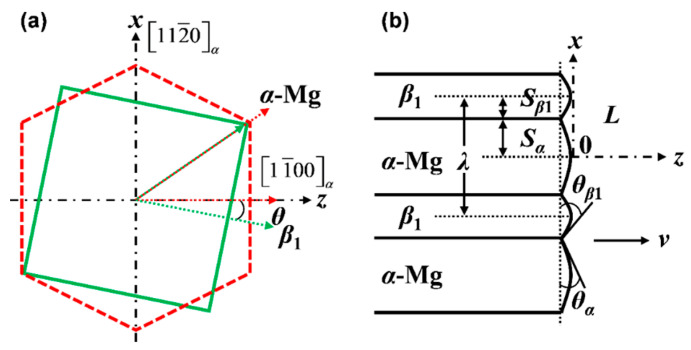
(**a**) Lattice vectors corresponding to the (011) *β*_1_ and (0001) *_α_* planes of *β*_1_ and *α*-Mg [34]; (**b**) schematic diagram of the lamellar eutectic structure at the eutectic dendrite tip region [35].

According to the references [19,30], the microstructure of as-cast Mg–Nd alloy consists of *α*-Mg matrix and divorced eutectic Mg_12_Nd (*β* phase). Other reports [31,32], however, have shown that the intermetallic phase formed in the eutectic was Mg_3_Nd (*β*_1_ phase) in as-cast Mg–Nd alloy, and the eutectic presented a lamellar structure. The *β* and *β*_1_ are both metastable precipitates; the *β* phase has a body-centered tetragonal structure (a = 1.031 nm, c = 0.593 nm) and the *β*_1_ phase has a face-centered cubic lattice (a = 0.74 nm). In this study, we mainly focused on the modelling and simulation for the eutectic formation of *α*-Mg matrix and *β*_1_-Mg_3_Nd precipitates. The detailed analytical model of lamellar and rod eutectic growth was first established by Jackson and Hunt (JH) in 1966 [33]. In the last decades, numerous researchers developed the JH model for investigating the eutectic growth mechanism. Although great progress has been made in the study of eutectic growth behavior, it must be pointed out that the knowledge and quantitative understanding of eutectic formation in the final solidification stage is still limited. Following the models established by JH and other researchers, a cellular automaton (CA) model was presented to investigate the evolution behavior of the *α*-Mg/*β*_1_ eutectic structure in Mg–Nd system alloys. Figure 1 shows the schematic diagrams of the lattice correspondence of *α*-Mg with *β*_1_ and the lamellar eutectic at the dendrite tip. In Figure 1b, *S*_α_ and *S_β_*_1_ represent half the widths of the *α* and *β*_1_ phases, respectively, and *v* is the growth velocity of the lamellar eutectic.

Solute diffusion is very important in determining eutectic growth. In this work, solute diffusion along *y* direction was ignored, and the solute field in liquid is given by:

(1) $$\frac{\partial^{2}C}{\partial x^{2}}+\frac{\partial^{2}C}{\partial z^{2}}+\frac{v}{D}\frac{\partial C}{\partial z}=0,$$

where *C* is the solute concentration in the liquid. The general periodic solution of the diffusion equation can be characterized by the following equation:

(2) $$C=C_{\infty }+\sum_{n=0}^{\infty }B_{n}e^{-\omega_{e}z}\cos\left(b_{n}x\right),$$

where *C*_∞_ is the solute concentration in the liquid far from the interface, *b_n_* = *n*π/(*S_α_* + *S_β_*_1_), and $\omega_{e}=\left(v/2D\right)+{\left[{\left(v/2D\right)}^{2}b_{n}^{2}\right]}^{1/2}$. The coefficient *B_n_* is obtained from the boundary conditions at the interface:

(3) $$-{\left(\frac{\partial C}{\partial z}\right)}_{z=0}=\left(\frac{v}{D}\right)C\left(x,0\right)\left(1-k_{\alpha }\right)\left(\alpha -\mathrm{Mg}\right),$$

(4) $$-{\left(\frac{\partial C}{\partial z}\right)}_{z=0}=-\left(\frac{v}{D}\right)[1-C\left(x,0\right)]\left(1-k_{\beta 1}\right)\left(\beta_{1}\;\mathrm{phase}\right),$$

where *C*(*x*, 0) is the interface concentration along the *α* or *β*_1_ phase, and *k_α_* and *k_β_*_1_ are the solute distribution coefficients in the *α* and *β*_1_ phases, respectively.

The growth kinetics are determined by the local undercooling (Δ*T*), which consists of thermal, constitutional and curvature effects that can be defined as:

(5) $$\Delta T=\Delta T_{e}+m_{i}\left(\omega -\omega_{e}\right)-\Gamma_{i}{\bar{K}}_{i},$$

where Δ*T_e_* is the metastable condition eutectic undercooling, *m_i_* is the liquidus slope (*i* denotes the *α* or *β*_1_ phase), *ω* is the concentration of the interfacial cell, Γ*_i_* is the Gibbs–Thomson coefficient of the *i* phase and ${\bar{K}}_{i}$ is the mean curvature of the interfacial cell, which can be approximately given by:

(6) $${\bar{K}}_{i}=\left[1-2\left(f_{s,i}+\sum_{j=1}^{N}f_{s,i}^{j}\right)/\left(N+1\right)\right]/\Delta x,$$

where $f_{s,i}$ and $f_{s,i}^{j}$ are the solid fraction of the *i* phase in the interfacial cell and its neighboring cells, respectively. *N* is the number of neighboring cells, which is equal to 8 in the actual calculations, Δ*x* is the cell size and the growth velocity, *v,* can be analytically expressed as:

(7) $$v=a_{i}\cdot {\left(\Delta T\right)}^{2},$$

where *a_i_* is the growth kinetic coefficient, and the value is 2.9 (μm·s^−1^·K^−2^) for the non-faceted *α* phase, while it is 5.8 (μm·s^−1^·K^−2^) for the faceted *β*_1_ phase [36]. The solid fraction of the *α* or *β*_1_ phase is calculated separately by the following equations:

(8) $$\Delta f_{s,\alpha }=v\cdot \Delta t_{n}/\Delta x,$$

(9) $$\Delta f_{s,\beta }=\cos\theta \cdot v\cdot \Delta t_{n}/\Delta x,$$

where Δ*t_n_* is the time step, and *θ* is the angle between the growth direction and the linking line, which is between the interfacial cell and the position of the nucleus of the *β*_1_ phase.

**Figure 2:**
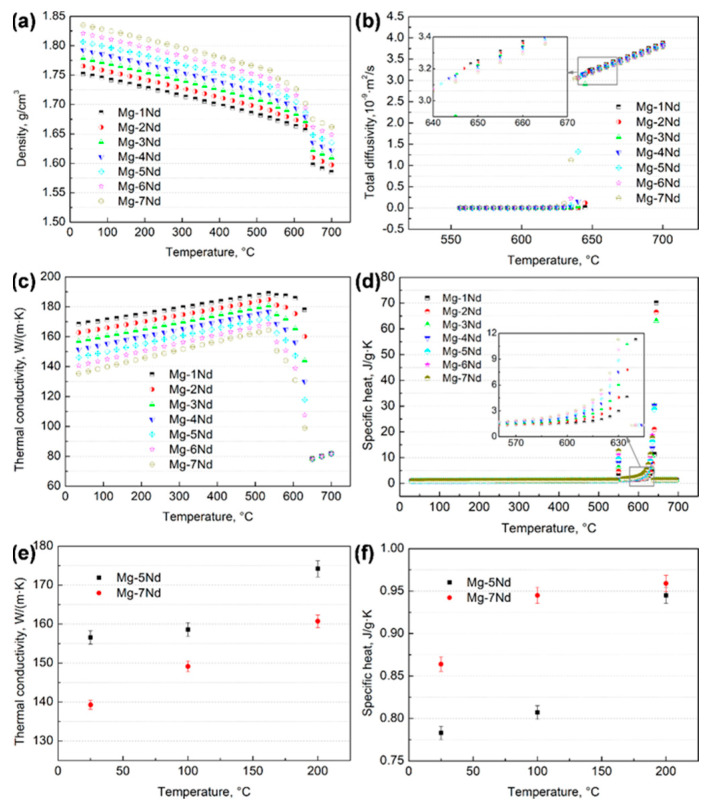
Thermophysical parameters of Mg–Nd system alloys: (**a**) density; (**b**) total diffusivity; (**c**,**e**) thermal conductivity, obtained by calculation and experiment; (**d**,**f**) specific heat, obtained by calculation and experiment.

Some thermophysical parameters of Mg–Nd system alloys were calculated by JmatPro software (CnTech Co., Ltd., Version 6.1, Shanghai, China), and they were compared with the experimental results, as shown in Figure 2. It can be seen that the density increases with the Nd additions, as the density of Nd is greater than that of Mg, and the values vary between 1.55 and 1.85 g/cm^3^. Meanwhile, in Figure 2c, the thermal conductivity decreases with increases in Nd content. The diffusion coefficient is shown in Figure 2b; the order of magnitude is ~10^−9^ in liquid, and it is ~10^−12^ in solid. In addition, the experimental measured results of the thermal conductivity and specific heat (shown in Figure 2e,f) fit well with the calculated results (Figure 2c,d). However, due to the limitation of JmatPro software, an optimized table look-up technique was adopted to obtain the parameters that were needed in the simulations, as shown in Table 2.

## 3. Results and Discussion

### 3.1. Microstructure and Precipitate Morphologies

**Figure 3:**
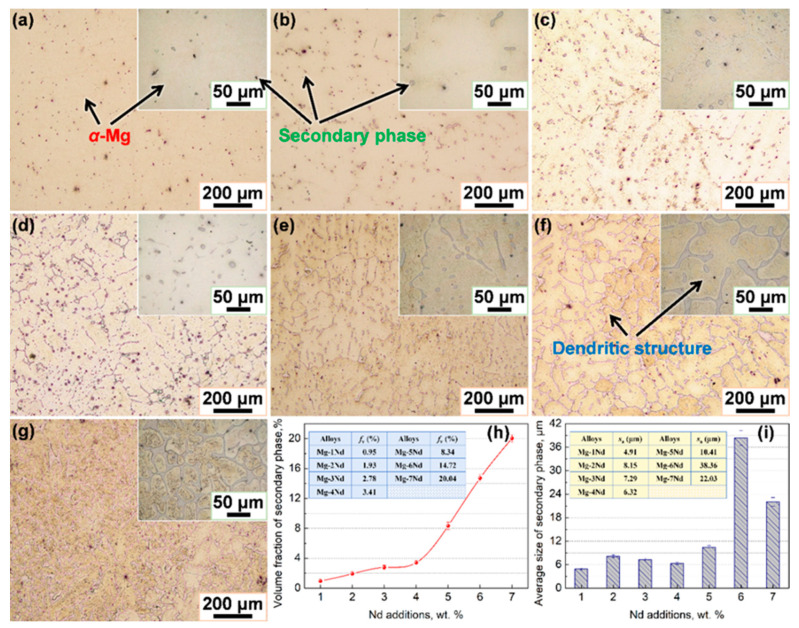
Low- and high-magnification optical micrographs of the microstructure and the secondary phase in different Mg–Nd system alloys: (**a**) Mg-1Nd; (**b**) Mg-2Nd; (**c**) Mg-3Nd; (**d**) Mg-4Nd; (**e**) Mg-5Nd; (**f**) Mg-6Nd; (**g**) Mg-7Nd; (**h**,**i**) the volume fraction and average size of secondary phase variation in Mg–Nd system alloys, respectively.

Low- and high-magnification optical micrographs of the microstructure and secondary phase morphologies in different Mg–Nd alloys are shown in Figure 3a–g, which presents the volume fractions and average sizes of the secondary phase variation in the Mg–Nd system alloys. It can be seen that the microstructure of Mg–Nd alloy consists of the dendritic *α*-Mg and a secondary phase (pointed by black arrows). The secondary phase is a Mg_12_Nd intermetallic compound, which is a metastable phase and always exists in the as-solidified alloys [19]. It is worth noting that the amount of secondary phase increases with Nd additions, and their morphologies change from blocky-shaped particles to a continuous network structure. According to previous reports [39,40,41] the maximum solubility of Nd in Mg is ~3.6 wt.% at the eutectic temperature (552 °C), while regarding the Mg–Nd binary phase diagram [42], Nd has the maximum solid solubility in Mg at ~548 ± 2 °C and almost zero at room temperature. Therefore, the solid solution of Nd in the Mg matrix is easy to saturate, and, as the Nd content increases, some of the Nd element does not go into the solid solution; it then exists in the form of a secondary phase, which always distributes discontinuously along the grain boundary. Due to the rapid growth of secondary phase along grain boundary, Nd-free bands are produced near the grain boundaries. In addition, it is observed that the microstructure of Mg–6Nd alloy consists of an obvious dendritic structure and network intermetallic phase (Figure 3f), which is consistent with those of a previous study [19]. The quantitative statistical results demonstrated that the volume fraction of the secondary phase increased from 0.95 % in Mg–1Nd to 20.04% in Mg–7Nd (Figure 3h). The average size of the secondary phase presented a relatively stable interval when Nd content was lower than 5 wt.%, and the value was approximately 4.91–10.41 μm, while the average size significantly increased to ~38.36 μm in Mg–6Nd alloy and decreased to ~22.03 μm when the content of Nd increased to 7 wt.% (Figure 3i). In addition, according to the study [19], the addition of Nd to Mg caused a significant improvement in the creep properties and the creep resistance increased with the increase in Nd addition, which is accounted for by the combination of precipitation and solid solution hardening.

**Figure 4:**
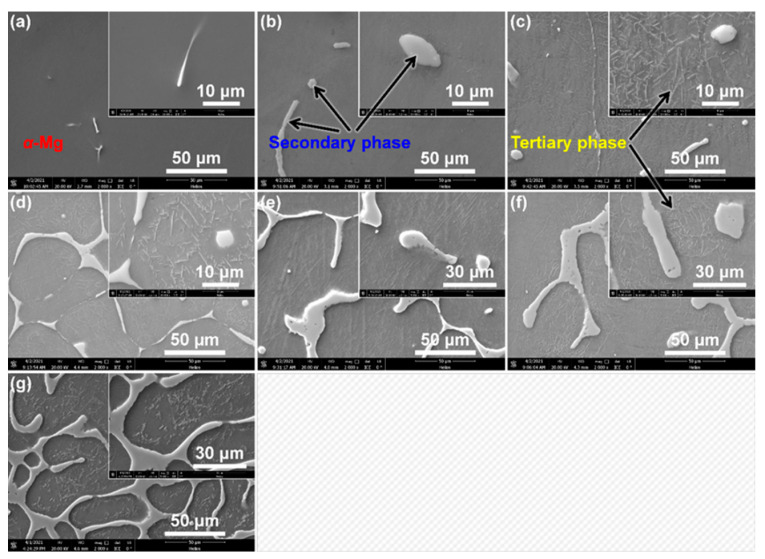
Low- and high-magnification SEM images of precipitate development in Mg–Nd system alloys: (**a**) Mg-1Nd; (**b**) Mg-2Nd; (**c**) Mg-3Nd; (**d**) Mg-4Nd; (**e**) Mg-5Nd; (**f**) Mg-6Nd; (**g**) Mg-7Nd.

In order to observe the precipitate development in Mg–Nd system alloys more clearly, FIB-SEM examinations were performed, as shown in Figure 4. SEM images are able to provide a good contrast between intermetallic or eutectic phases and the *α*-Mg matrix. The contrast among different phases in the microstructure was exploited to obtain different grayscale images from which the intermetallic or eutectic phases and *α*-Mg matrix were determined. It can be seen that the lower Nd content alloys (Mg–1Nd and Mg–2Nd in Figure 4a,b) are mainly composed of two different phases, i.e., *α*-Mg and a secondary phase (*β* phase, Mg_12_Nd), which is in agreement with the OM observations mentioned above. The formation of a secondary phase is due to the non-equilibrium solidification of Nd in *α*-Mg being oversaturated, and part of the Nd forms divorced eutectic *β*-Mg_12_Nd instead of precipitating in the as-cast alloys. With the increasing of Nd additions, however, a number of tiny precipitates form from the oversaturated *α*-Mg matrix, temporarily named the tertiary phase (marked by black arrows in Figure 4c,f). Based on the previous reports [28,31], the tertiary phase might be a *β*_1_ phase (Mg_3_Nd), and the microstructure of this alloy, in fact, contains a eutectic mixture of the intermetallic phase and *α*-Mg. It is noted that the result is quite different from previous studies [6,19,30], which have only found the divorced Mg_12_Nd intermetallic in the as-cast binary Mg–Nd alloys. In addition, according to the Mg–Nd binary phase diagram [42], a *β*_1_-Mg_3_Nd phase tends to form in the high-Nd-content alloys, for example, in the master alloy with the Nd content over 20 wt. %. Nevertheless, at the relatively low content of Nd, Mg3Nd could also form at a higher cooling rate [31], in an extruded state Mg–Nd alloy [32] or after high-pressure torsion [43]. The *β*_1_ is also a metastable phase and presents a zigzag structure that seems to have precipitated homogeneously in the *α*-Mg matrix. The morphology and distribution of the *β*_1_ phase changed with Nd contents, and the morphology of *β*_1_ phase in the lower Nd content alloys is obviously different from that in the Mg–7 Nd alloy (Figure 4g). The variation may be due to the different interaction mechanism between strain and dislocation in different Nd addition alloys.

### 3.2. Chemical Composition and Phase Characterization

**Figure 5:**
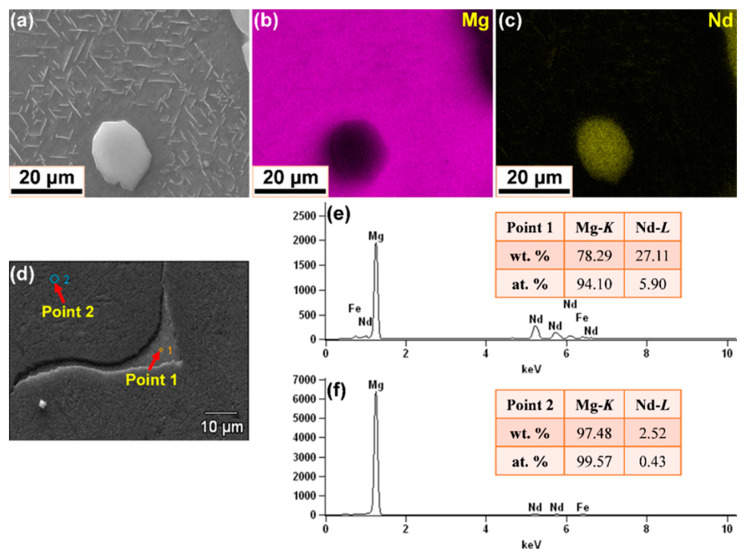
Micrographs and chemical composition analysis of Mg–3Nd alloy: (**a**–**c**) BSE image and corresponding elemental mappings; (**d**–**f**) SEM image and point EDS results of the secondary phase and Mg matrix.

**Figure 6:**
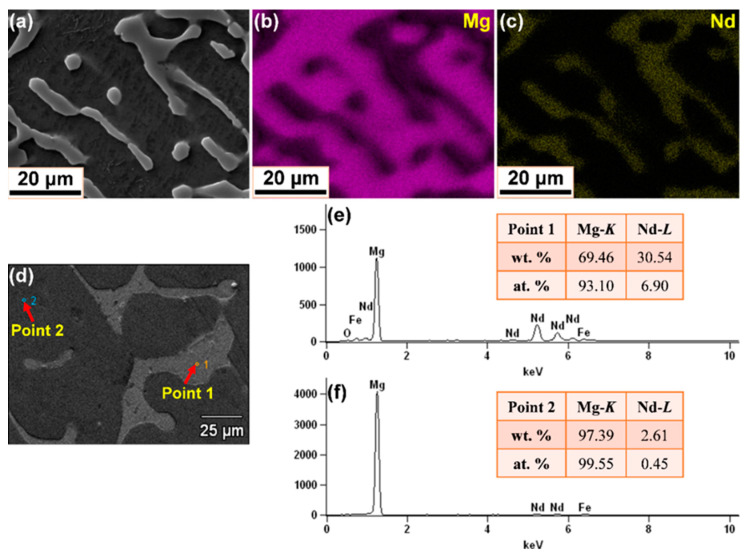
Micrographs and chemical composition analysis of Mg–6Nd alloy: (**a**–**c**) BSE image and corresponding elemental mappings; (**d**–**f**) SEM image and point EDS results of the secondary phase and Mg matrix.

Figure 5 and Figure 6 present the micrographs and chemical composition analysis of Mg–3Nd and Mg–6Nd alloys, respectively. The elemental mappings in Figure 5b,c indicate that the secondary phase is rich in Nd, and the matrix is mainly composed of Mg element, where the lighter pixels represent a higher element content, and the darker ones illustrate a lower element content. Nevertheless, it is hard to identify the element distribution of the tertiary phase in elemental mappings, and a similar phenomenon could be found in Figure 6b,c. In addition, the impurity elements, such as Fe and P, were not detected, which was probably due to artifacts, such as a peak overlap in the EDS mappings. In addition, with the increasing Nd content, the enrichment of Nd presents semi-continuously intermixed with the Mg matrix. In order to obtain further insight into the distribution of chemical elements, EDS analysis was pursued. Figure 5d–f and Figure 6d–f display the point EDS results in the secondary phase (light-grey region, Point 1 in the figures) and Mg matrix (dark-grey region, Point 2 in the figures). Since the point EDS actually measured the composition in a region larger than the spots in both Mg–3Nd and Mg–6Nd alloys, the analysis included a portion of the matrix and a number of particles. It can be seen that the elemental composition at Point 1 and Point 2 in Mg–3Nd alloy were approximately 78.29 wt.% Mg and 27.11 wt.% Nd and 97.48 wt.% Mg and2.52 wt.% Nd, respectively, while it was about 69.46 wt.% Mg and 30.54 wt.% Nd and 97.39 wt.% Mg and 2.61 wt.% Nd in Mg–6Nd alloy. The amount of Nd in the matrix presents a relatively small change between Mg–3Nd and Mg–6Nd alloys, and this is because the additional amount of Nd is more than its solubility limit in both alloys. It should be noted here that the point EDS analysis shows that Fe element was detected in both alloys, which most likely derived from the raw materials or the tools used for melting and casting, such as the steel mold and stirring tool.

**Figure 7:**
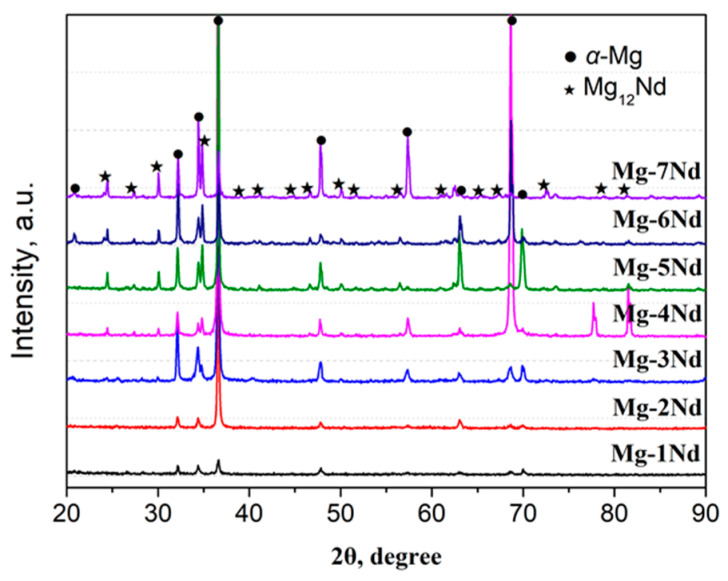
X-ray diffraction patterns of Mg–Nd system alloys.

Figure 7 displays the X-ray diffraction patterns of Mg–Nd system alloys. It can be seen that all the peaks are indexed as arising from two different phases, i.e., *α*-Mg and *β*-Mg_12_Nd, which is consistent with the OM observations in Figure 2. The *β*-Mg_12_Nd is a metastable phase, and its amount increases with the Nd levels, based on the statistical results mentioned above. Other reports [18,44,45], however, indicated that the *β*_e_-Mg_41_Nd_5_ phase could also be detected in the as-extruded Mg–Nd alloys and at high temperatures. Actually, the sequence and type of precipitation are affected by the alloy state, solidification condition, heat treatment process, etc. In the present work, the *β*-Mg_12_Nd phase easily formed in the as-cast Mg–Nd alloys under the determined solidification condition. Regrettably, the tertiary phase was not detected in all Mg–Nd system alloys; this may be because the amount of tertiary phase was less and the volume was small in the as-cast Mg–Nd system alloys. According to the analysis and deduction above in this work, the tertiary phase (*β*_1_-Mg_3_Nd) is a metastable eutectic phase that likely precipitates from the supersaturated *α*-Mg during cooling after casting. In addition, the metastable intermetallic phases (*β* or *β*_1_) would transform to the equilibrium phase at a certain temperature during heating, such as under the subsequent solution and aging conditions.

### 3.3. Simulation of Eutectic Growth and Development

**Figure 8:**
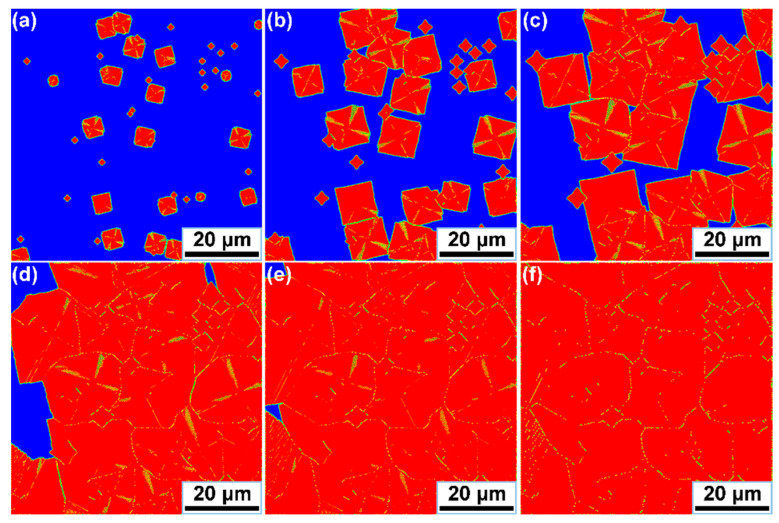
Simulation results of *α*-Mg/*β*_1_-Mg_3_Nd eutectic growth for Mg-3Nd alloy at various times: (**a**) 0.27 s; (**b**) 0.53 s; (**c**) 0.82 s; (**d**) 1.15 s; (**e**) 1.53 s; (**f**) 2.21s.

In this section, to provide further investigation of the evolution behavior of the eutectic in Mg–Nd system alloys, the proposed CA model was applied to simulate the *α*-Mg/*β*_1_-Mg_3_Nd eutectic growth and morphologies. The corresponding parameters used in the simulation cases are shown in Figure 2 and Table 2, some of which vary with the solidification conditions. As a representative, Mg-3Nd alloy was selected to illustrate the growth process of the eutectic structure, and the simulation results are shown in Figure 8. A square-shaped domain was used for the simulation cases consisting of 400 × 400 cells. No mass flux conditions were imposed at the calculation boundaries, and the temperature was constant. The total eutectic nuclei number was equal to 50 in the simulation case, with randomly assigned locations and preferential growth orientations. The red color in Figure 8 represents the *α*-Mg dendrites, the yellow color represents the *β*_1_-Mg_3_Nd precipitates and the blue color represents the liquid phase. Initially, the nucleated grains are randomly distributed in the calculation domain, and the *α*-Mg dendrites start to develop along the preferential growth orientations that present a petaliform morphology. It can be seen that the growth of *α*-Mg dendrites is accompanied by the nucleation and growth of *β*_1_-Mg_3_Nd precipitates and eventually forms the eutectic structure. The *β*_1_-Mg_3_Nd precipitates tend to be suppressed by the faster-growing *α*-Mg dendrites, resulting in the discontinuous growth of *β*_1_. Actually, the eutectic growth mainly depends on the solute distribution. With the continuous solidification of the liquid alloy, excess solute is rejected into the melt, and the interaction effects of the solute field between the adjacent *α*-Mg dendrites are intensified (Figure 8b,c). The *β*_1_-Mg_3_Nd will absorb amounts of solute from the surrounding liquid and nucleate at grain boundaries. It can be seen that *β*_1_-Mg_3_Nd precipitates at grain boundaries present the zigzag shape (Figure 8d,e), which is consistent with the experimental observations in Section 3.1. Figure 8f shows the final morphology of the *α*-Mg/*β*_1_-Mg_3_Nd eutectic structure. It can be found that most *β*_1_-Mg_3_Nd precipitates distribute along the grain boundaries and form interconnected networks. In addition, it is also noticeable that the *β*_1_-Mg_3_Nd precipitates formed at the *α*-Mg dendritic arms are eliminated or decreased; this is probably because *β*_1_ is a metastable phase, and it will be dissolved as the temperature drops lower. The divorced eutectic *β*-Mg_12_Nd also has an important influence on the formation of *β*_1_-Mg_3_Nd at grain boundaries, which are limited by the proposed CA model; however, the microstructure in the simulation results do not contain the divorced eutectic *β*-Mg_12_Nd.

**Figure 9:**
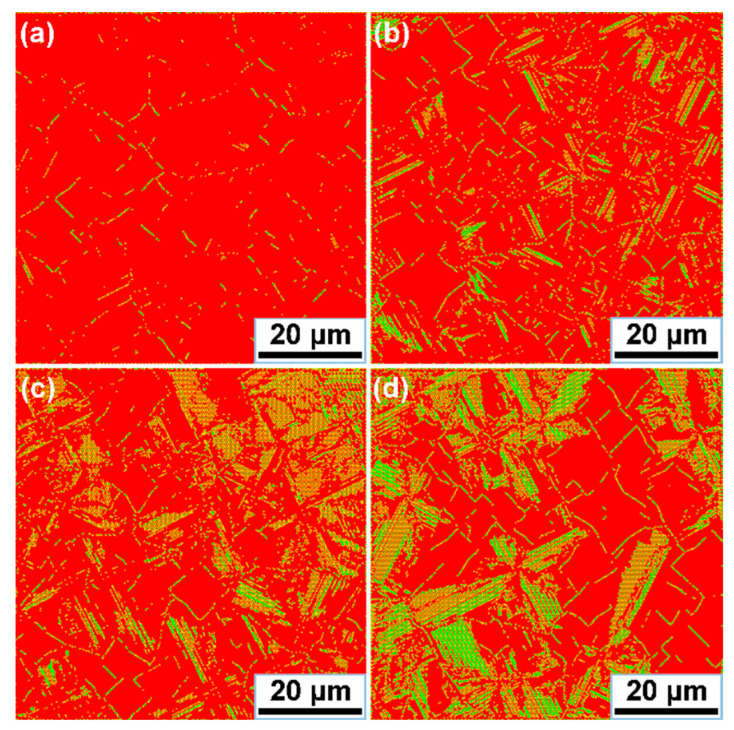
Simulation results of *α*-Mg/*β*_1_-Mg_3_Nd eutectic morphologies for Mg–Nd system alloys: (**a**) Mg-4Nd; (**b**) Mg-5Nd (**c**) Mg-6Nd; (**d**) Mg-7Nd.

In order to investigate the effect of Nd additions on the *α*-Mg/*β*_1_-Mg_3_Nd eutectic, simulation cases with various Nd contents were performed, and the simulation results are shown in Figure 9. In the Mg–Nd system alloy, *α*-Mg is the faster growing phase, and the volume fraction of *β*_1_-Mg_3_Nd in the eutectic structure is very small; thus, it is difficult to describe this intermetallic phase when the content of Nd is lower. Therefore, Mg–Nd system alloys containing 4 wt.%, 5 wt.%, 6 wt.% and 7 wt.% were selected for the simulations. It can be observed that the eutectic morphologies with different Nd contents have a similar characteristic, consisting of *α*-Mg dendrites, zigzag-shaped *β*_1_-Mg_3_Nd at grain boundaries and rod-like *β*_1_-Mg_3_Nd in the dendritic regions. As the Nd content increases, the *α*-Mg dendrite is refined, and the amount of the eutectic structure is clearly promoted. In addition, as the eutectic fraction increases with the Nd content, the morphologies undergo a prominent transition from isolated zigzag or rod-like shapes to interconnected networks. The proposed model incorporated several aspects, including growth algorithms, kinetics and solute diffusion, to achieve the eutectic growth mechanism for Mg–Nd system alloys. It can be seen that the simulation and experimental results presented a good agreement, indicating that this model can successfully reproduce the eutectic growth of Mg–Nd system alloys. As known to us, in addition to the influence of alloying elements, other factors such as the cooling rate and undercooling also have significant effects on the eutectic growth. However, due to the limitation of the established model and technical algorithm, this study only focused on the *α*-Mg/*β*_1_-Mg_3_Nd eutectic development with the Nd additions. In our following work, we will make an effort to investigate the precipitation mechanism of *β*_1_-Mg_3_Nd to further improve the simulation precision.

## 4. Conclusions

In this work, experimental and computational methods were used to investigate the microstructure and precipitate development in as-cast Mg–Nd system alloys. OM, SEM and XRD analyses were performed, and a eutectic growth model was established. The main conclusions are shown as follows:

(1) The Mg–Nd alloys with different Nd contents (1, 2, 3, 4, 5, 6 and 7 wt.%) were prepared, and the microstructure and precipitate development in Mg–Nd system alloys was observed by OM and SEM techniques. According to the OM micrographs, the microstructure of Mg–Nd alloy consisted of *α*-Mg dendrites and the secondary phase, and the amount of secondary phase presented an increasing trend with the increasing Nd additions. SEM images indicated that a tertiary phase precipitated from the oversaturated *α*-Mg matrix in the higher Nd content alloys, and this is different from the previous studies, which only found a divorced eutectic Mg_12_Nd in as-cast Mg–Nd alloys.

(2) Chemical composition and phase characterizations were performed by EDS and XRD methods, respectively. The point EDS results indicated that the Nd content in the *α*-Mg matrix presented a relatively small change between Mg–3Nd and Mg–6Nd alloys, as the additional amount of Nd was more than the solubility limit in both alloys. The secondary phase was further confirmed to be Mg_12_Nd by XRD analysis. Regrettably, the tertiary phase was not detected in all Mg–Nd system alloys; this may be because the amount of tertiary phase was less and the volume was small in the as-cast alloys.

(3) An effective CA model was explored, with the advantage of describing the time-dependent *α*-Mg/*β*_1_-Mg_3_Nd eutectic growth. The *α*-Mg/*β*_1_-Mg_3_Nd eutectic growth in Mg–3Nd alloy was simulated using the proposed CA model, and the simulated results revealed that the growth of *α*-Mg dendrites was accompanied by the nucleation and growth of *β*_1_-Mg_3_Nd precipitates and eventually formed the eutectic structure. In addition, eutectic morphologies for Mg–Nd system alloys with different Nd contents were also simulated, and the results indicated that the *α*-Mg dendrite was refined and the amount of *α*-Mg/*β*_1_-Mg_3_Nd eutectic was promoted with an increase in the Nd content.

## Figures and Tables

**Table 1:** Chemical compositions of Mg–*x* Nd alloys.

Alloys	Designed Compositions		Analyzed Compositions	
	Nd (wt.%)	Mg	Nd (wt.%)	Mg
Mg–1 Nd	1	Bal.	0.95	Bal.
Mg–2 Nd	2	Bal.	1.98	Bal.
Mg–3 Nd	3	Bal.	3.10	Bal.
Mg–4 Nd	4	Bal.	3.92	Bal.
Mg–5 Nd	5	Bal.	4.85	Bal.
Mg–6 Nd	6	Bal.	5.89	Bal.
Mg–7 Nd	7	Bal.	6.92	Bal.

**Table 2:** Thermophysical parameters of Mg–Nd alloy from Refs [31,36,37,38].

Definition and Units	Values
Eutectic temperature (°C)	552
Eutectic composition (wt.%)	33
Liquid slope *m_α_* (°C/wt.%)	−5.1
Liquid slope *m_β_*_1_ (°C/wt.%)	13.2
Gibbs–Thomson coefficient of *α*-Mg (m·K)	6.2 × 10^−7^
Gibbs–Thomson coefficient of the *β*_1_ phase (m·K)	1.7 × 10^−7^
Solute distribution coefficient of *α*-Mg	0.4
Solute distribution coefficient of the *β*_1_ phase	0.113

## Data Availability

The data presented in this work are available on request from the corresponding authors.
